# Urethroplasty Using A Turnover Flap for Correctıon of Problematıc Urethrocutaneous Fıstula: A Case Report

**Published:** 2017-05

**Authors:** Husnu Tokgoz, Gulsum Tetik, Soner Yalcinkaya, Ali Yildiz, Murat Savas

**Affiliations:** Department of Urology, Antalya Training and Research Hospital, Antalya, Turkey

**Keywords:** Buck’s fascia, Fistula, Hypospadias, Transverse turnover flap

## Abstract

Urethral fistula formation after urethroplasty for hypospadias is a frequent complication. Repeated failures can occur even after multiple attempts at repair. A surgical procedure is described for a problematic resistant urethrocutaneous fistula (UF) with the transverse turnover flap using the Buck’s fascia of the corpus cavernosum. A 23-year-old male was admitted to our hospital with recurrent coronal UF. We placed a suprapubic catheter in the bladder and operated the patient with the flap technique combined with glanuloplasty. In 3rd month follow up, the patient had no fistula with normal voiding.

## INTRODUCTION

Hypospadias is one of the most common congenital urogenital anomalies in the male population. Prevalence ranges between 0.5-8 per 1000 live births.^1^ Surgery is the only treatment modality for the disease. However, urethrocutaneous fistula (UF) is the most important complication related to hypospadias surgery.^2^ Because, due to the lack of healthy surrounding tissue, it is difficult to manage a UF case. Despite various types of UF management techniques have been described, most popular way seems to perform a three-layer reconstruction. Inner urethral closure surrounded by tissue flaps and outer closure with the cutaneous coverage was mostly performed. But, in resistant cases with repeated surgical failures, there is no consensus.^3^ Herein, we used a turnover flap lifted from the Buck’s fascia of the corpus cavernosum and placed over the fistula site in combination with glanuloplasty. 

## CASE REPORT

A 23-year-old male patient was admitted to our clinic with coronal fistula. On his physical examination, UF fistula was present in coronal sulcus. The patient had no signs of chordee and associated urogenital anomaly. Fibrosis was present at the operation site related to previous operations. During micturition, all the urine was coming from the UF site. In the past, the patient said that he was operated five times. In 1999, he had distal hypospadias surgery. After 1 month of follow up, UF was developed. From 1999 to 2013, he had primary UF closure operations in different hospitals (2003 and 2006). In 2013, he had his fourth operation with the use of his buccal mucosal greft. 

**Fig. 1:**
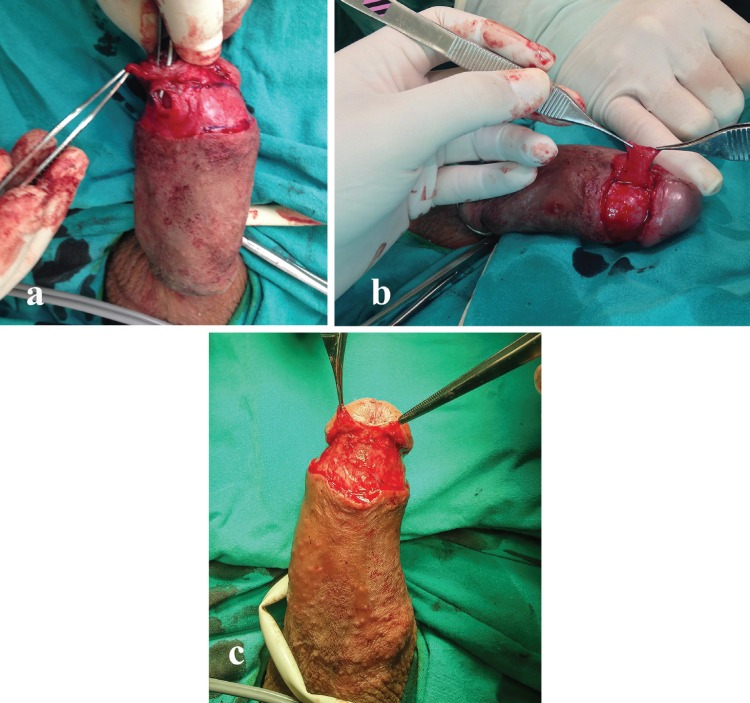
Images showing the (a) localization of urethrocutaneous fistula (b) transverse turnover flap lifted from the Buck’s fascia of the left corpus cavernosum (c) placement of the flap over the fistula via 6-0 monocryl suture

After the failure with buccal mucosal graft, he had his fifth operation as primary UF closure with the use of surrounding soft tissues as a flap between the inner urethral and outer skin layers. However, he had UF at the same region, 3 weeks after the operation. We decided to operate the patient. Under spinal anesthesia, we put a suprapubic tube in order to prevent urine leakage and infection caused by urethral catheter. The incision was performed in circumcision line, a transverse turnover flap taken from the Buck’s fascia of the left corpus cavernosum was lied over urethra in order to close the 5x5 mm fistula site (Figure 1).

Meanwhile, glanuloplasty was performed and skin was closed over the flap. The patient was discharged on first postoperative day with oral antibiotics, and the suprapubic tube was removed on postoperative 7th day. After a 3-month follow-up, the patient had normal voiding with no recurrence of UF. In terms of erection, full erection without a chordee was demonstrated in 3rd month follow up visit.

## DISCUSSION

Among the different surgical procedures advocated in hypospadias repair, urethrocutaneous fistula remains the most frequent encountered complication and a serious problem, even in experienced surgical hands. The reported rate of fistula formation after hypospadias repair varies between 1% and 91% in literature.^4^^,^^5^ Avoidance of overlapping suture lines, use of fine scalpel for skin incision, minimal tissue trauma by use of fine forceps or retracters may prevent urethrocutaneous fistula formation following hypospadias surgery.^6^^,^^7^

Because of the scar formation after each surgical procedure for the re-correction of UF, the success rates decrease dramatically after each attempt for surgical repair. Our case had multiple attempts for the repair, so we defined the case as a problematic UF. For this reason, we need to bring a healthy, vascular and resistant tissue (tissue tension must be high enough to prevent damage to neourethra caused by high-pressure urine flow during voiding) between the inner urethral and outer skin layer. As the urethral calibration was good, we did not make a central incision of the urethral plate, but we trimmed the fistula border to remove the scar tissue around the fistula site.

Most vascular tissue around the fistula was Buck’s fascia and we lifted a vascular flap from Buck’s fascia. In coronal UF cases, we also believe that, glanuloplasty is necessary for an effective outer skin layer closure, so, we also performed glanuloplasty. In current literature, although various modifications have been defined, to the best of our knowledge, our case was the first which used isolated turnover flap taken from the Buck’s fascia of the corpus cavernosum to the fistula site and, combined with glanuloplasty. Bae* et al.* preferred to use a turnover flap that was lifted from penile ventral skin and named the procedure as modified Mathieu procedure.^8^

Huang *et al.* used onlay island flaps and reported a 12,6% of UF rate.^9^ Choi *et al.* tried to correct 9 cases with UF who had previous one- or two-stage repair with tubularized incised-plate urethroplasty (TIPU) and used dartos fascia-reinforced flaps.^10^ They tubularized the plate and covered by dartos and prepuce. However, another group of plastic surgeons preferred to use scrotal septal flaps.^11^ Firstly, they destroyed the hair follicles in the donor site 2 months before the operation. Eventually, they covered the urethral defect with a longitudinal flap isolated from the scrotal septum. 

In conclusion, as the Buck’s fascia has a good blood supply and tissue tension, it can be transferred as a transverse turnover flap over urethra. The procedure is simple and should be helpful in problematic UF cases with repeated re-correction failures.

## CONFLICT OF INTEREST

The authors declare no conflict of interest.
